# Multicellular detachment generates metastatic spheroids during intra-abdominal dissemination in epithelial ovarian cancer

**DOI:** 10.1038/s41388-018-0317-x

**Published:** 2018-05-23

**Authors:** Sara Al Habyan, Christina Kalos, Joseph Szymborski, Luke McCaffrey

**Affiliations:** 1 Rosalind and Morris Goodman Cancer Research Centre, McGill University, Montreal, QC H3A 1A3 Canada; 2 Division of Experimental Medicine, McGill University, Montreal, QC H4A 3J1 Canada; 3 Gerald Bronfman Department of Oncology, McGill University, Montreal, QC H4A 3T2 Canada

## Abstract

Ovarian cancer is the most lethal gynecological cancer, where survival rates have had modest improvement over the last 30 years. Metastasis of cancer cells is a major clinical problem, and patient mortality occurs when ovarian cancer cells spread beyond the confinement of ovaries. Disseminated ovarian cancer cells typically spread within the abdomen, where ascites accumulation aids in their transit. Metastatic ascites contain multicellular spheroids, which promote chemo-resistance and recurrence. However, little is known about the origin and mechanisms through which spheroids arise. Using live-imaging of 3D culture models and animal models, we report that epithelial ovarian cancer (EOC) cells, the most common type of ovarian cancer, can spontaneously detach as either single cells or clusters. We report that clusters are more resistant to anoikis and have a potent survival advantage over single cells. Using in vivo lineage tracing, we found that multicellular spheroids arise preferentially from collective detachment, rather than aggregation in the abdomen. Finally, we report that multicellular spheroids from collective detachment are capable of seeding intra-abdominal metastases that retain intra-tumoral heterogeneity from the primary tumor.

## Introduction

Metastasis is responsible for more than 90% of all the cancer-associated deaths. Intra-abdominal metastasis is frequently observed in gastrointestinal and gynecological cancers, in which cells disseminate and grow in the abdominal cavity. Intra-abdominal dissemination is particularly prevalent in epithelial ovarian cancer (EOC), the most lethal gynecological cancer, with fewer than 30% of patients surviving 5 years after diagnosis [1, 2]. The majority (85–90%) of EOCs express epithelial markers (E-cadherin and cytokeratin) and acquisition of epithelial characteristics may be necessary for cell transformation during ovarian cancer initiation [3]. The epithelial state of tumors at secondary sites is less clear, with reports of retention or loss of E-cadherin in metastases, implicating tumoral heterogeneity and epithelial plasticity in this process [3–7].

Ovarian cancer dissemination is associated with malignant ascites, which is present in a third of patients at diagnosis and almost all patients at recurrence, and is considered a major source of chemo-resistance, recurrence, and mortality [1, 8]. Malignant ascites contains disseminated tumor cells as single cells, or more commonly, as multicellular spheroids, in a complex fluid that constitutes a pro-tumorigenic environment [1, 5, 7, 9, 10]. Spheroids are considered bona fide metastatic units that can attach to the mesothelium and invade the extracellular matrix during dissemination [1, 9, 11–14]. Current models propose a multi-step process for intraperitoneal metastasis that includes: (1) shedding from the primary tumor; (2) evading anoikis; (3) formation of spheroids; and (4) peritoneal implantation and outgrowth [15, 16]. In comparison to the late stages of peritoneal implantation, the early stages of shedding, survival, and spheroid formation remain poorly understood. Despite the critical importance of spheroids during intra-abdominal dissemination, a long-standing question is how spheroids form. One hypothesis proposes that multicellular spheroids arise from single cells aggregating within the abdomen [13, 17]. An alternative possibility is that cells detach as groups that form spheroids. Here we report that spheroids predominantly arise from multicellular detachment from the primary tumor and are responsible for intraperitoneal metastasis. Furthermore, we report that detaching spheroids can maintain phenotypic heterogeneity of the primary tumor during dissemination.

## Results and discussion

### Epithelial cancer cells spontaneously detach in culture

**Fig. 1:**
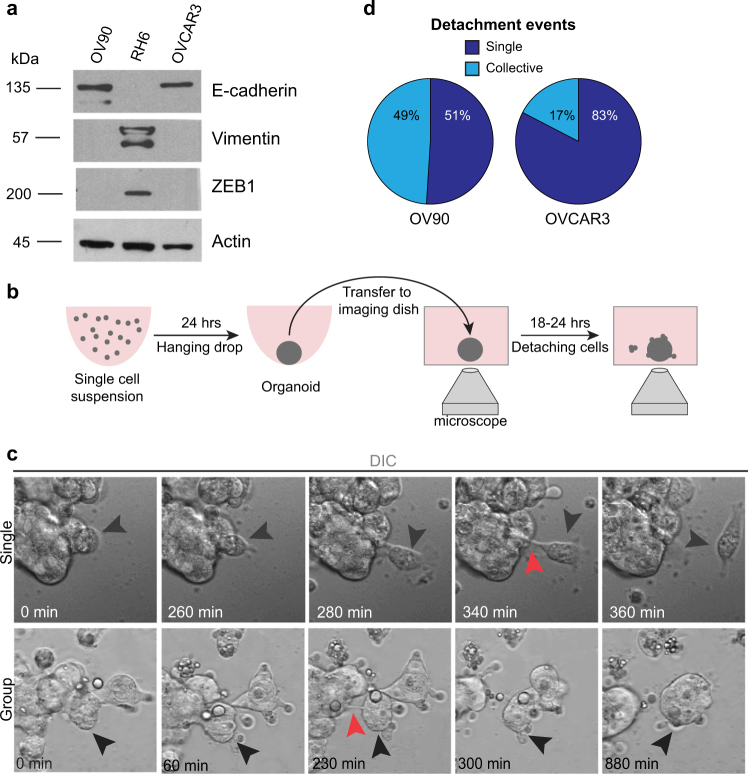
Epithelial ovarian cancer cells (EOC) spontaneously detach as single cells or clusters. **a** EOC cell lines (OV90 and OVCAR3) were immunoblotted for epithelial and mesenchymal markers. RH6 lysates were included as a control for mesenchymal markers [18]. Human NIH:OVCAR3 cells were obtained from ATCC. Human OV90 (originally isolated from malignant ascites from a patient with adenocarcinoma) and RH6 were obtained from Dr. Patricia Tonin (McGill University) [18]. OVCAR3 and OV90 were originally isolated from malignant ascites from patients with ovarian adenocarcinoma, have p53 mutations, and exhibit genomic features similar to high-grade serous ovarian carcinoma [27–29]. Cell lines were maintained at low passage numbers, routinely tested for the absence of *Mycoplasma* contamination, and were validated by STR profiling. OV90 cells were cultured in OSE, 10% FBS, P/S, 10 mM HEPES buffer, and 4 mM of l-glutamine. RH6 cells were cultured in the same medium supplemented with 4 mg/mL Hygromycin. OVCAR3 cells were cultured in RPMI 1640, 20% FBS, P/S, and 0.01 mg/mL insulin. Western blots were performed as previously described [30]. **b** Overview of experimental design for imaging detachment of cells from 3D organoids. Organoids were generated in hanging drops [31] then imaged without embedding in extracellular matrix. **c** DIC images showing a representative time-lapse series of single cell and collective cell detachment events for OV90 cells. Cells (1 × 10^6^ cells/mL) were incubated as hanging droplets for 24 h to generate organoids. Organoids were transferred to polyhema (Sigma) coated 8-well coverglass chambers (LabTek) in growth media containing 20% methocel (Sigma). Live-imaging of organoids was performed using a LSM700 confocal microscope with a 20 × 0.8NA objective lens and ZEN software (Zeiss). Between 30 and 45 organoids were imaged in three independent experiments. Images were captured every 10 min for 15–22 h in an environmental chamber (37 °C, 5% CO_2_). Movies were manually inspected to detect detachment events. Black arrowheads show detaching cells. Red arrowheads show stalks connecting detaching cell(s) from the parental group. **d** Quantification of single cell and collective detachment events. Time-lapse videos were analyzed using ZEN software (Zeiss). OV90, *n* = 85; OVCAR3, *n* = 23

During routine culture of epithelial ovarian cancer cells (OV90 and OVCAR3), we observed detached cells, frequently present as spheroids, floating in the culture medium. Using calcein and eithidium homodimer to detect live and dead cells, respectively, we observed that many of the detached cells were alive (Fig. S1a–c). Since cell detachment has been linked to reduced cell–cell adhesion mediated by loss of E-cadherin and acquisition of a mesenchymal phenotype, we examined expression of E-cadherin, ZEB1, and Vimentin expression by western blot (Fig. 1a). This revealed that both OV90 and OVACR3 cells retained epithelial characteristics in culture. RH6 cells, a mesenchymal derivative of OV90, were used as a control [18]. This indicates that epithelial ovarian cancer cells can spontaneously detach and survive in culture.

To further explore early events during dissemination of epithelial ovarian cancer cells we performed live-imaging to directly visualize detachment events. For this we used 3D culture environment that more accurately mimics the architecture of a tumor, where cells detach from other cells, rather than the plastic surface of a dish. Many 3D cultures are embedded in a hydrogel matrix, which mimics the extracellular matrix and stroma that cells invade through. However, unlike some other solid cancers that invade through the stroma to leave the primary site during hematological metastasis, ovarian cancer cells shed from the surface of the tumor epithelium into the abdominal cavity [2]. Therefore, we used the hanging-drop method to generated a 3D organotypic model that was free-floating, rather than embedded in extracellular matrix, and established novel imaging conditions to observe cells detaching from the cellular surface into free space using time-lapse microscopy (Fig. 1b, Videos S1–2). Under our organotypic culture conditions, OV90 cells form tight organoids, whereas OVCAR3 cells formed loose organoids, as reported previously [19]. We observed a total of 85 detachment events from OV90 organoids, of which 49% were by groups of two or more cells (Fig. 1c, d). In both single and multicellular detachment events, we observed cellular stalks that connect the leaving cell(s) with the parental cell mass (Fig. 1c, d). This indicates that for both single and collective detachment, the final step is a single-cell detachment event. Detachment events from OVCAR3 organoids were less frequent than OV90, and 17% (4/23) events detached as groups of two or more cells (Fig. 1d, S2a). Therefore, epithelial cancer cells can detach as either single cells or collectively as groups of cells in vitro.

### Spheroids in ascites are generated from collective detachment

**Fig. 2:**
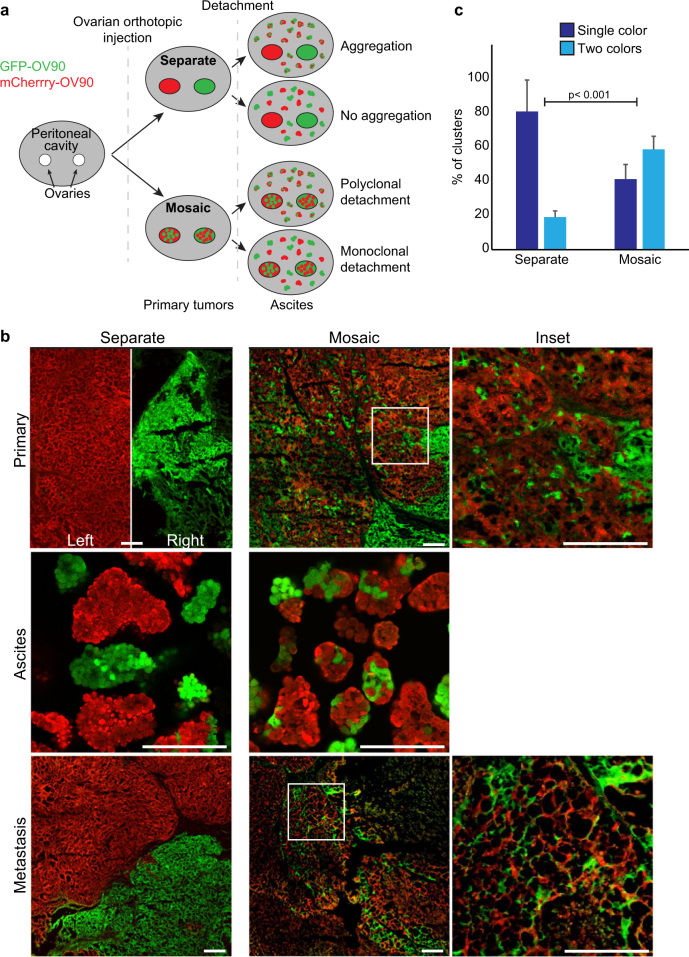
Multicellular clusters in ascites arise from collective detachment in vivo. **a** Overview of experimental design. Lentivirus was produced in HEK293LT cells in accordance with McGill University biohazard regulations and as described previously [30]. The lentivector pWPI was a gift from Didier Trono (Addgene plasmid # 12254) and was modified to replace GFP with mCherry. GFP- and mCherry-expressing human OV90 cells were either injected into contralateral ovaries (“Separate”) or were mixed within the same ovary (“Mosaic”). Female (8–12-week-old) athymic nude mice (Crl:NU (NCr)-Foxn1nu; Charles River) were housed at the GCRC animal facility and all procedures were performed following ethics approval in accordance with the animal care guidelines at the Animal Resource Centre of McGill University. For othotopic ovarian injections, 7.5 × 10^5^ OV90 cells as a single-cell suspension in 100% Geltrex (Invitrogen) in a 10 μL volume were injected into the ovary. We did not observe leakage from the injection site. Ovarian tumors formed in ~6 weeks and ascites were detected in 8–10 weeks; *n* = 5 mice per condition. G*Power2 was used to estimate sample sizes for moderate to large effects sizes (>0.5). Ascites (volume range: 0.5–2 mL) and omental metastases (range: 3–12 macro-metastases) were observed in all mice. Animals that did not develop primary tumors, ascites, and metastases were excluded. Animals from the “Separate” group that developed only one primary tumor on either side were excluded. No specific randomization method was used. **b** Fluorescent images showing GFP- and mCherry-labeled cells in primary tumors, ascites, and peritoneal wall metastases. Primary and secondary tumors were resected from euthanized mice and fixed in 4% PFA (pH 7.2–7.4) for 24 h at 4 °C. Ascites were collected using a 23G needle, and the cellular fraction was embedded in 1.5% agarose in PBS after PFA fixation. Tissues and ascites spheroids were embedded in OCT compound (Tissue Tek) and cryosectioned. PFA fixation preserves fluorescent protein brightness [32], which was visualized directly using a LSM700 confocal microscope with a 20 × 0.8NA objective lens and ZEN software (Zeiss). Scale bars = 200 μm. **c** Quantification of spheroid colors in ascites. Individual spheroids were scored as expressing GFP or mCherry (Single color) or both GFP and mCherry (two colors). Images were contrast-enhanced using Fiji/ImageJ software (National Institute of Health, NIH), with uniform parameters applied across comparative images. Investigators were not blinded to the experimental groups. Differences in the distribution between separate and mixed conditions were assessed using a *χ*^2^ test; *p* < 0.001; error bars, s.d.

To determine whether cell clusters in ascites in vivo arise from aggregation or collective detachment, we performed lineage tracing. We generated OV90 cells expressing either GFP or mCherry and transplanted these orthotopically into contralateral ovaries of the same mouse (“Separate”), to generate one red and one green primary tumor (Fig. 2a). If spheroids in ascites arose from aggregation, then we expected the clusters would be composed of both red and green cells. Conversely, if spheroids were generated in the absence of aggregation, then we expected them to be a single color. We validated that seeding of primary tumor from the opposite color was negligible in this assay, since primary tumors remained a single color (Fig. 2b). Strikingly, we observed that about 80% of spheroids isolated from ascites were composed of single color cells, indicating that intra-abdominal mixing is an infrequent event (Fig. 2c).

Once we established that intra-abdominal aggregation is not the major mechanism to generate spheroids, we tested whether they were polyclonal, which would indicate collective detachment. For this we generated mice where GFP and mCherry-expressing cells were mixed and injected into the same ovary, creating mosaic tumors (Fig. 2b, “Mosaic”). Under these conditions, we observed mosaic (red and green) spheroids in ~60% of spheroids isolated from ascites. The proportion of mixed colored spheroids likely underestimates the frequency of multicellular detachment events in vivo, since multicellular clusters of the same color may also detach. Single-colored clusters from Mosaic primary tumors may also arise from detaching single cells that grow into clusters in ascites. Together, these data support a model by which cells detach as clusters from the primary tumor to generate spheroids in metastatic ascites with a minor contribution from aggregation. We were unable to distinguish if infrequent cases of aggregation arose from clustering of single cells or smaller multicellular spheroids.

Spheroids are thought to be responsible for metastatic spread in ovarian cancer [1, 2]. We therefore asked whether single spheroids gave rise to individual metastatic lesions within the abdominal cavity, or whether multiple spheroids contributed to individual metastatic lesions. We reasoned that if single spheroids gave rise to individual metastatic lesions, we would observe single color metastases in mice with “Separate” green and red primary tumors. Alternatively, if multiple spheroids contributed to individual metastatic lesions, we would expect mixed GFP/mCherry lesions under these experimental conditions. We observed that intra-abdominal metastases were single colored, indicating that individual metastatic lesions arose from single spheroids (Fig. 2b, c; see “Separate”). In contrast, in mice-expressing mosaic GFP/mCherry primary tumors and spheroids, the secondary lesions were also predominantly mosaic with extensive intermixing of GFP and mCherry (Fig. 2b, c; see “Mosaic”). This supports a model in which spheroids are metastatic units, and that metastatic sites are formed from single spheroids that detached as groups of cells from the primary tumor.

Our results are consistent with genetic clonal mapping of disseminated ovarian cancers in patients. McPherson et al. performed phylogenetic analysis of multiple intraperitoneal sites from individual patients, and reported that most sites were phylogenetically pure or highly related from a single phylogenetic clade [20]. However, each patient also had rare sites that were polyphyletic clones from multiple distinct clade [20]. Our data indicate that individual sites arise from single spheroids and provide a mechanism to explain divergent clonal dissemination events. We show that the majority of spheroids in ascites arise from collective dissemination of neighboring cells, which can explain the origin of the phylogentically pure or related disseminated sites. We also identified a small fraction of spheroids that form from intraperitoneal mixing, which can explain the origin of infrequent sites of polyphyletic clones observed in human ovarian cancer patients.

### Detaching clusters have a survival advantage compared to detaching single cells

**Fig. 3:**
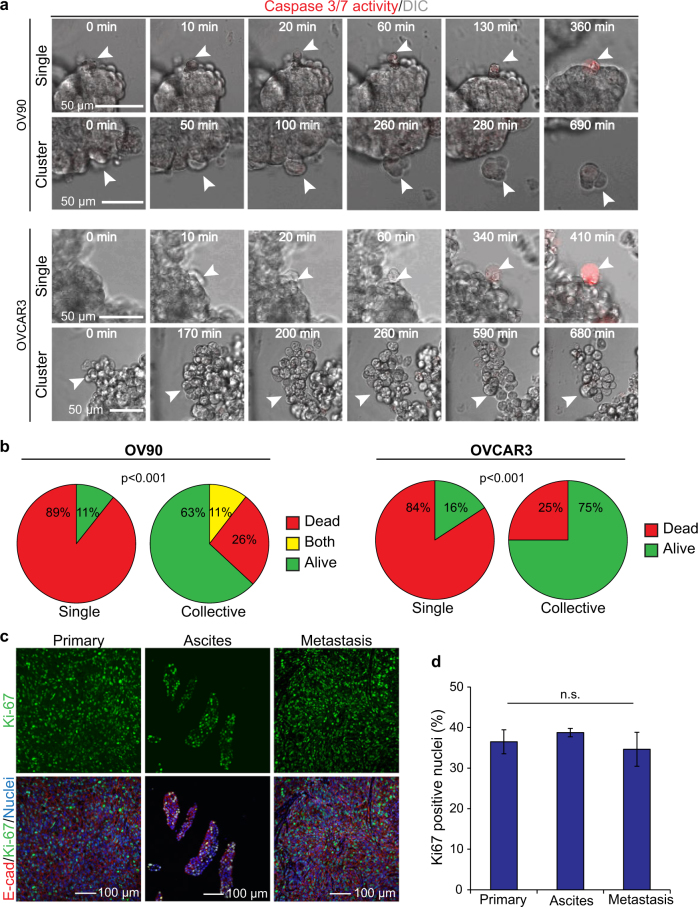
Cells that detach collectively have a survival advantage over detaching single cells and are proliferative. **a** DIC/fluorescence images from representative time-lapse series of single and cluster detachment for OV90 and OVCAR3 cells. Cells were treated with 2 µM CellEvent^TM^ Red dye (Invitrogen), a fluorescent marker for active Caspases 3 and 7. **b** Quantification of apoptosis events in single cells and clusters during detachment from organoids in OV90 (*n* = 47; *p* < 0.001) and OVCAR3 (*n* = 23; *p* < 0.001) cells. Some clusters were predominantly alive, but contained at a single dead cell, and were labeled as “Both”. **c** Immunofluorescent images of primary, ascites, and peritoneal wall metastases stained for the proliferation marker Ki67 and counter-stained for E-cadherin. Paraffin-embedded tissue was processed by the GCRC Histology Core Facility, and antigen-retrieval and immunostaining of tissue sections were performed as described previously [30]. Antibodies and concentrations are listed in Supplementary Table 1. **d** Quantification of Ki67 positive cells from the indicated sites; *n* = 3; *p* > 0.05; error bars, s.d.

Our in vitro organotypic cultures demonstrated that epithelial ovarian cancer cells were able to efficiently detach as either single cells or clusters, while our in vivo data suggested a preference towards multicellular detachment. Loss of cell contact with other cells or extracellular matrix can induce apoptosis which must be overcome for cancer cells to survive and disseminate [21]. We therefore predicted that detaching epithelial clusters might have a survival advantage over single cells. To test this, we labeled cells with a fluorescent apoptosis reporter of Caspase 3/7 activity and performed live-imaging to track detachment events and their survival fate in vitro. Whereas most detaching single cells (84–89%) underwent rapid apoptosis as they left the organoid, the majority of detaching clusters avoided anoikis (Fig. 3a, b; Videos S3–6). Curiously, we observed a small number of detaching clusters from OV90 organoids that had a single cell undergo apoptosis, whereas the majority of cells in the cluster were alive. The cause of this is not known, but may arise from stress induced on individual cells within the cluster during detachment.

### Clusters in ascites contain proliferating cells

In patients, a wide range of sizes of spheroids are observed [22]. We also observed a wide range of sizes of clusters in ascites, ranging from less than 20 cells (small) to over 100 cells (large) (Fig. S3a). Although some large clusters may arise during detachment, we tested whether they had proliferative capacity that could enable to smaller clusters to grow in ascites. Consistent with this idea, we observed that 38% of cells in clusters were positive for the proliferation marker Ki67 (Fig. 3c, d). This proliferative capacity was similar to that observed in both the primary and secondary tumors, indicating that proliferation is preserved throughout metastasis in multicellular clusters. Therefore, spheroids in ascites can arise from detaching cell clusters or, rarely, individual cells that retain proliferative properties of the primary tumor.

### Spheroids maintain phenotypic heterogeneity during dissemination

**Fig. 4:**
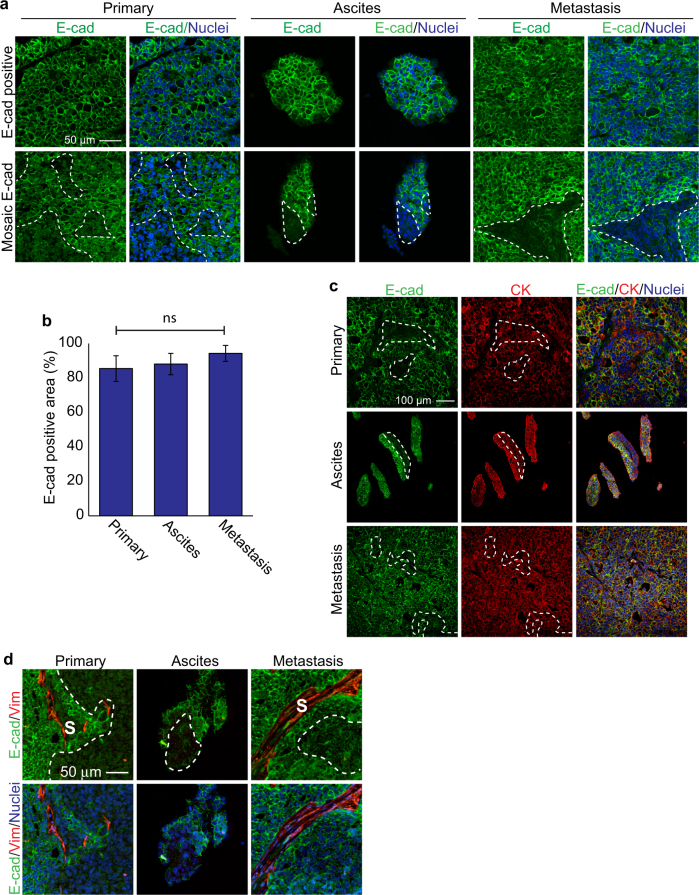
Intra-tumor heterogeneity is maintained in multicellular clusters in ascites. **a** Immunofluorescence images of primary tumor, ascites, and peritoneal wall metastases stained for E-cadherin. **b** Quantification of E-cadherin-positive areas in primary, ascites, and peritoneal wall metastases, *n* = 6; *p* > 0.05; error bars, s.d. E-cadherin-positive regions were classified as having >50% circumferential E-cadherin immunoreactivity surrounding each cell and were pan-cytokeratin positive. Regions were considered E-cadherin negative when two or more adjacent cells had no detectable or incomplete (<50%) circumferential E-cadherin and regions were pan-cytokeratin positive. **c** Immunofluorescence images of tumor sections stained for E-cadherin and pan-cytokeratin. **d** Immunofluorescence images of tumor sections stained for E-cadherin and Vimentin. S indicates Vimentin-positive stroma. White dotted lines show boundaries between E-cad^+^ and E-cad^−^ populations

E-Cadherin heterogeneity is well documented in primary ovarian cancers and ascites from patients, and may assist late events in metastasis like peritoneal attachment and growth [3, 23]. This prompted us to investigate whether phenotypic heterogeneity present in the primary tumor could be maintained during dissemination through collective events. We observed in vivo that regions of primary tumor were E-cadherin positive, with small patches of E-cadherin negative cells (Fig. 4a, b). Further analysis demonstrated that these cells were positive for pan-cytokeratin and negative for Vimentin in the tumor compartment, suggesting an overall epithelial phenotype. Strong Vimentin staining was present in the stroma, acting as a positive control for this marker (Fig. 4c, d). Importantly, the E-cadherin heterogeneity was maintained in ascites, with some spheroids containing both E-cadherin positive and negative sub-populations, and was also present in secondary lesions on the peritoneal wall, with no statistical difference in the proportion of E-cadherin positive and negative zones. Therefore, our results support a model by which collective detachment can maintain phenotypic heterogeneity in ascites, and subsequently in secondary tumors.

Although the interplay between specific ovarian cancer cell populations will be the focus of future studies, our results provide a conceptual model by which intra-tumor heterogeneity may be preserved through collective metastasis. Indeed, our lineage tracing indicate that individual metastatic lesions arise from single spheroids, and therefore maintaining interactions between sub-populations may provide an advantage during dissemination. In support of this idea, spheroids engineered in vitro to contain mosaic E-cadherin positive and E-cadherin negative populations displayed enhanced proliferation compared the pure populations [23]. Moreover, an association between intra-tumor heterogeneity and clinical outcomes like resistance, relapse, and mortality has been established across cancers, and can result from cooperation among sub-clones [24]. In addition to promoting survival of cells, we propose that detachment of multicellular clusters could have additional benefits during metastasis and seeding secondary sites. Tumorigenic potential can be influenced by non-cell autonomous cooperation between tumor cell populations. For example, breast cancer tumor-initiating cells communicate with mesenchymal-like niche cells to promote tumorigenicity [25]. Interactions between cancer cell populations in ovarian cancer have not been thoroughly investigated, but ovarian cancer cells do benefit from non-cell autonomous interactions with other cell types. For example, the presence of a cellular microenvironment was sufficient to enable ovarian cancer cells to form tumors in xenograft mouse models that were unable to do so when the cancer cells were injected alone [26]. Moreover, Yin et al. [17] reported that ovarian cancer spheroid formation is enhanced by tumor-associated macrophages, which provide growth support to the epithelial tumor cells through EGFR signaling to form aggregates from single cells. An important distinction with these previous experiments and ours is that previous experiments were conducted with single-cell suspensions injected intraperitoneally in mice, and therefore model mechanisms relating to how single cells aggregate to form spheroids once detached, but do not address the origin of spheroids from solid tumors [17]. Our studies provide strong evidence that collective detachment is an important mechanism that generates multicellular spheroids, but do not exclude a possible role for macrophages in supporting their growth.

In summary, we demonstrate that spheroids in metastatic ascites can arise from collective dissemination, which promotes resistance to apoptosis, maintains phenotypic heterogeneity.

## Electronic supplementary material

Video S1

Video S2

Video S3

Video S4

Video S5

Video S6

Supplementary Material
