# Supplementary material for: Multicellular detachment generates metastatic spheroids during intra-abdominal dissemination in epithelial ovarian cancer
Source: Oncogene. 2018 May 23;37(37):5127–35. doi: 10.1038/s41388-018-0317-x (PMC6137025; doi:10.1038/s41388-018-0317-x)
Supplement: Supplementary file 7 — Supplementary Material [file 41388_2018_317_MOESM7_ESM.pdf]

Supplemental Material

Title:

Collective cell detachment forms multicellular spheroids in ascites that maintain intratumoral heterogeneity during ovarian cancer metastasis.

Authors:

Sara Al Habyan, Christina Kalos, Joseph Szymborski, Luke McCaffrey[1]

Table S1: Primary antibodies used in this study

Figure S1: Epithelial ovarian cancer cells can spontaneously detach as single cells or clusters in 2D culture.

Figure S2: Epithelial ovarian cancer cells can spontaneously detach as single cells or clusters.

Figure S3: Quantification of the size distribution of multicellular clusters isolated from ascites in mice

Video S1: Collective detachment of OV90 cells.

Video S2: Single cell detachment of OV90 cells.

Video S3: Single cell detachment of OV90 cells with fluorescent caspase marker.

Video S4: Collective detachment of OV90 cells with fluorescent caspase marker.

Video S5: Single detachment of OVCAR3 cells with fluorescent caspase marker.

Video S6: Collective detachment of OVCAR3 cells with fluorescent caspase marker.

Supplementary Table 1: Primary antibodies used in this study.

Primary antibody	Application	Dilution	Incubation	Company	Catalogue number
**Actin**	WB	1:500	O/N in 5% milk	Santa Cruz	sc-8432
**E-cadherin**	WB	1:5000	O/N in 5% milk	CST	3195
**ZEB1**	WB	1:500	O/N in 5% BSA	CST	3396
**Vimentin**	WB	1 in 5000	O/N in 5% milk	CST	5741
**Pan CK**	WB	1:2000	O/N in 5% milk	CST	4545
**ZO-1**	WB/IF	1:1000	O/N in 5% milk (WB) or 5% GS (IF)	CST	8193
**E-cadherin**	IF	1:500	O/N in 5% GS	BD transduction	610181
**Ki67**	IF	1:500	O/N in 5% GS	Abcam	ab15580

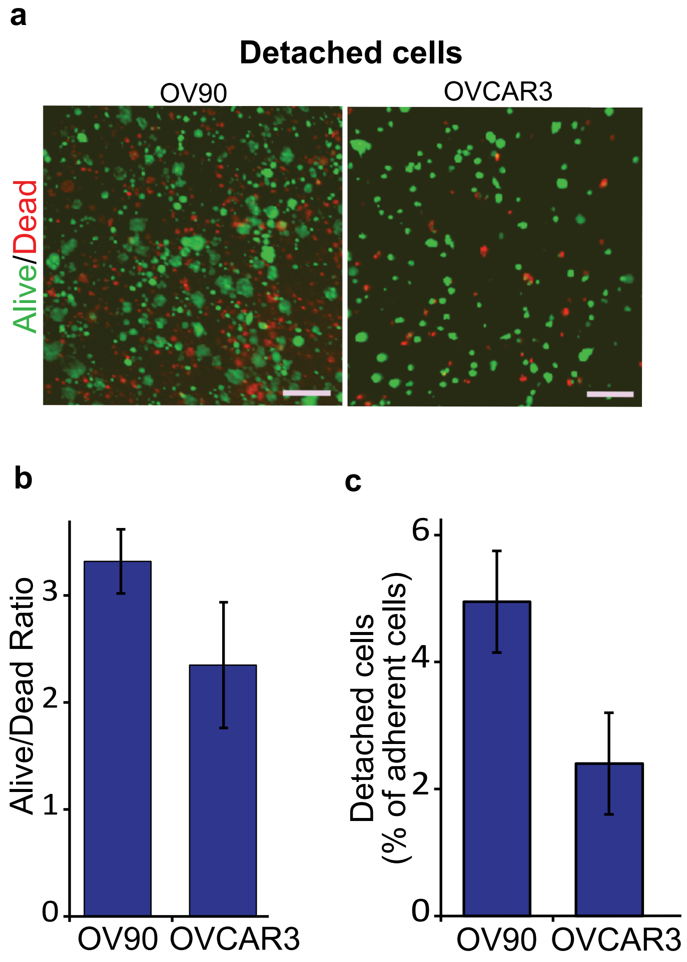

Supplementary Figure S1 (related to Figure 1)
Epithelial ovarian cancer cells can spontaneously detach as single cells or clusters in 2D culture. A, fluorescence images showing spontaneously detached cells from OV90 and OVCAR3 monolayers stained with calcein (green) and ethidium homodimer (red) to identify live and dead cells, respectively. B, quantification of the proportion of attached and detached cells in the indicated cell lines. C, quantification of the live/dead ratios of detached cells (n=3).

Al habyan et al.

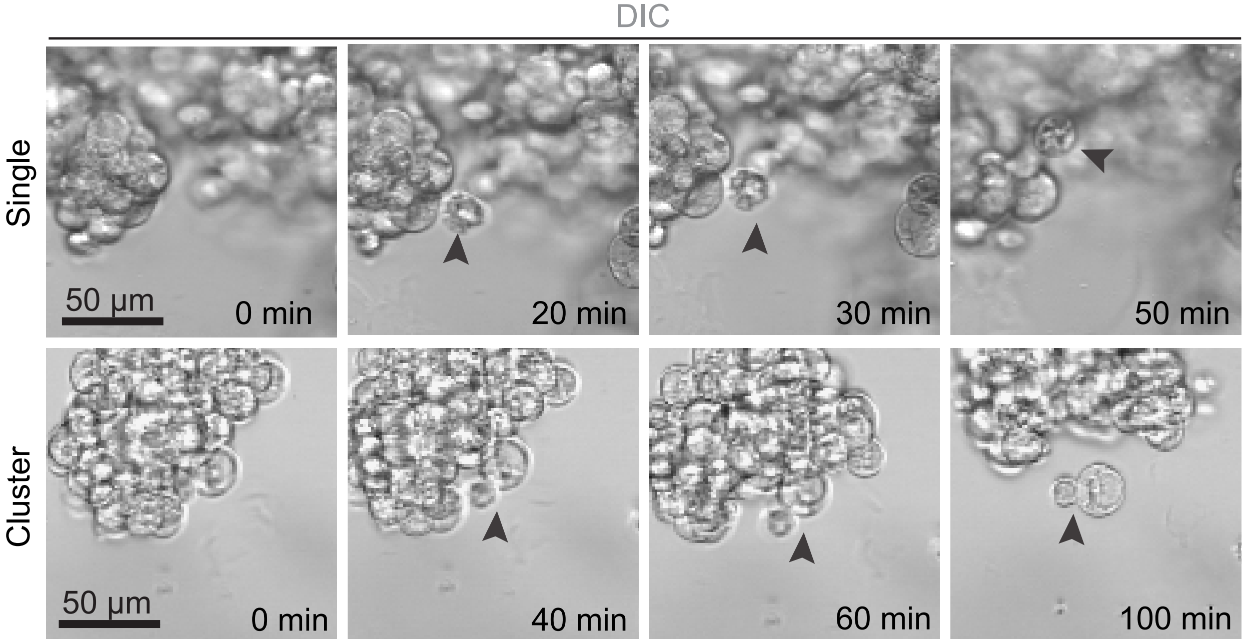

Supplementary Figure S2 (related to Figure 1)
Epithelial ovarian cancer cells can spontaneously detach as single cells or clusters. DIC images showing a representative time-lapse series of single cell and collective cell detachment events for OVCAR3 cells.

Al habyan et al.

a

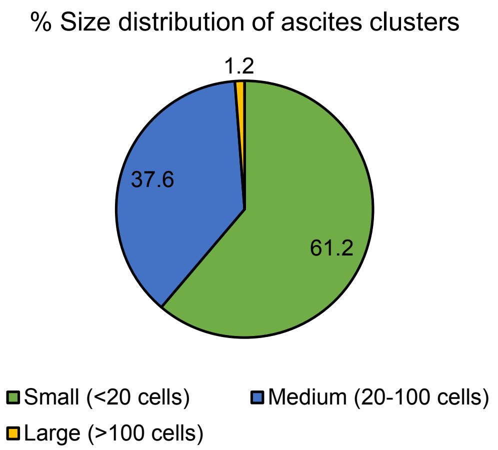

Supplementary Figure S3 (related to Figure 3)
Quantification of the size distribution of multicellular clusters isolated from ascites in mice.
